# The impact of Parkinson’s disease-associated gut microbiota on the transcriptome in *Drosophila*

**DOI:** 10.1128/spectrum.00176-23

**Published:** 2023-09-27

**Authors:** Xin Liu, Meng Yang, Runzhou Liu, Fan Zhou, Haibing Zhu, Xiaoyun Wang

**Affiliations:** 1 South China Normal University-Panyu Central Hospital Joint Laboratory of Translational Medical Research, Guangzhou Panyu Central Hospital, Guangzhou, China; 2 Guangdong Provincial Key Laboratory of Insect Developmental Biology and Applied Technology, School of Life Sciences, South China Normal University, Guangzhou, China; 3 Department of Psychiatry, Guangzhou Panyu Central Hospital, Guangzhou, China; Brigham Young University, Provo, Utah, USA

**Keywords:** Parkinson’s disease, gut microbiota, *Drosophila melanogaster*, fecal microbiota transplantation, transcriptome

## Abstract

Parkinson’s disease (PD) is a common neurodegenerative disease in middle-aged and elderly people, and many studies have confirmed that the disorder of gut microbiota is involved in the pathophysiological process of PD. However, the molecular mechanism of gut microbiota in regulating the pathogenesis of PD is still lacking. In this study, to investigate the impact of PD-associated gut microbiota on host transcriptome, we established various PD models with fecal microbiota transplantation (FMT) in the model organism *Drosophila* followed by integrative data analysis of microbiome and transcriptome. We first constructed rotenone-induced PD models in *Drosophila* followed by FMT in different groups. Microbial analysis by 16S rDNA sequencing showed that gut microbiota from PD *Drosophila* could affect bacterial structure of normal *Drosophila*, and gut microbiota from normal *Drosophila* could affect bacterial structure of PD *Drosophila*. Transcriptome analysis revealed that PD-associated gut microbiota influenced expression patterns of genes enriched in neuroactive ligand-receptor interaction, lysosome, and diverse metabolic pathways. Importantly, to verify our findings, we transplanted *Drosophila* with fecal samples from clinical PD patients. Compared to the control, *Drosophila* transplanted with fecal samples from PD patients had reduced microbiota *Acetobacter* and *Lactobacillus*, and differentially expressed genes enriched in diverse metabolic pathways. In summary, our results reveal the influence of PD-associated gut microbiota on host gene expression, and this study can help better understand the link between gut microbiota and PD pathogenesis through gut-brain axis.

**IMPORTANCE:**

Gut microbiota plays important roles in regulating host gene expression and physiology through complex mechanisms. Recently, it has been suggested that disorder of gut microbiota is involved in the pathophysiological process of Parkinson’s disease (PD). However, the molecular mechanism of gut microbiota in regulating the pathogenesis of PD is still lacking. In this study, to investigate the impact of PD-associated gut microbiota on host transcriptome, we established various PD models with fecal microbiota transplantation in the model organism *Drosophila* followed by integrative data analysis of microbiome and transcriptome. We also verified our findings by transplanting *Drosophila* with fecal samples from clinical PD patients. Our results demonstrated that PD-associated gut microbiota can induce differentially expressed genes enriched in diverse metabolic pathways. This study can help better understand the link between gut microbiota and PD pathogenesis through gut-brain axis.

## INTRODUCTION

The human gut has tens of trillions of microorganisms, including more than 1,000 species of bacteria. The number of genes of the microorganisms were 100 times larger than the human genome, and a balanced gut microbiota is critical for maintaining general health (1). More importantly, studies in the past 15 years have demonstrated gut microbiota as one of the key regulators of brain function, and have led to the appreciation of the importance of microbiota-gut-brain axis (2). Mechanistically, the highly bidirectional communication between the brain and the gut can be markedly influenced by the microbiota through integrated immunological, neuroendocrine, and neurological processes. At the molecular level, gut microbiota and its relevant metabolites interact with the host via a series of biochemical and functional pathways, thereby affecting host homeostasis and health (3).

Parkinson’s disease (PD) is the second most common neurodegenerative disease. Worldwide, about 3 million people suffer from the debilitating symptoms of PD involving motor deficits such as akinesia, muscular rigidity, tremor, slowness of movement, difficulty in walking, and gait. Also, PD is a multifactorial disease with less than 10% of cases being related to hereditary, therefore the pathogenesis of PD has a strong environmental component (4, 5). Recently, the gut microbiota-brain axis is gaining increasing attention by researchers who investigate the biological and physiological basis of psychiatric, neurodevelopmental, age-related, and neurodegenerative disorders (6). Current evidence from different clinical studies has revealed that gut microbiota composition is altered in PD patients and is related to clinical phenotypes (7), suggesting that gut microbiota plays a crucial role in PD. However, the pathogenesis of gut microbiota-mediated PD is still unclear and the microbiota-based clinical treatments are also limited. Fecal microbiota transplantation (FMT) is the most effective method to reconstruct the gut microbiota of PD patients (8). Although current research on FMT in the treatment of PD is not in-depth, FMT has been considered as a possible intervention for gut microbiota disturbance in the treatment of PD.

Fruit flies *Drosophila* are capable of performing complex motor behaviors such as walking, climbing, and flying, and the brains of fruit flies are functionally similar to humans (9). Basic cellular processes and many genes as well as signaling pathways are conserved between fruit flies and humans (10). The availability of genetic tools, the rapid growth and reproduction, the low cost, and the convenience to maintain in the laboratory make *Drosophila* an ideal model system to address biological questions relevant to human health (11, 12). The establishment of a toxin-based PD model in fruit flies has made an important contribution to our understanding of the disease (13 – 15). In such a scenario, *Drosophila* has emerged as a valuable model for studying mechanisms of human neurodegenerative diseases including PD. In this study, we constructed a rotenone-induced PD model in *Drosophila* followed by fecal microbiota transplantation, and investigated the impact of gut microbiota on host transcriptome by 16S rDNA sequencing and RNA sequencing. Our findings were verified by transplanting *Drosophila* with fecal samples from clinical PD patients. Our findings demonstrate the influence of PD-associated microbiota on host transcriptome in *Drosophila* and provide new insights into the mechanism of microbiota colonization in PD at the molecular level.

## RESULTS

### Rotenone-induced Parkinson’s disease models in *Drosophila* and FMT experiments

**Fig 1:**
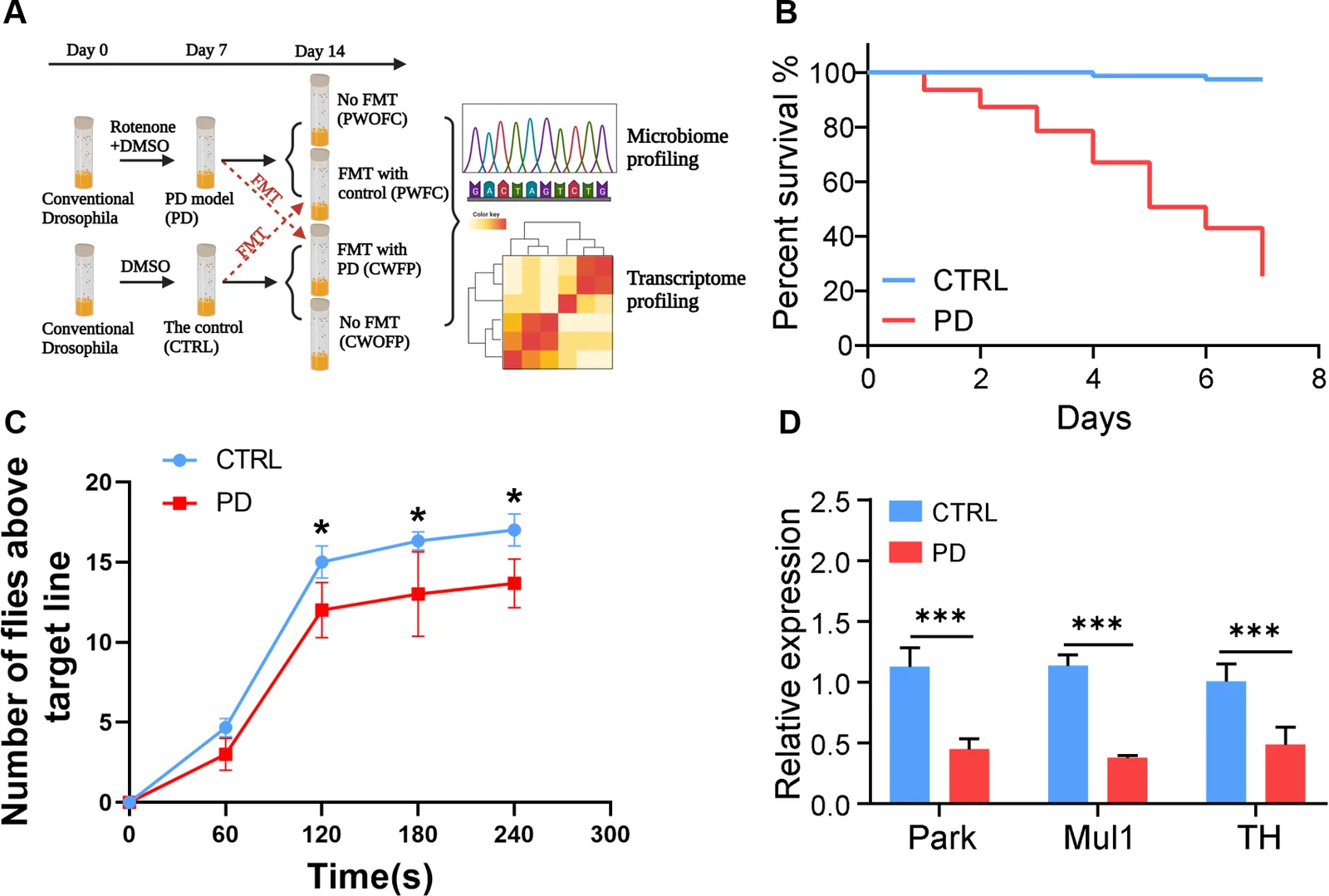
Phenotypic analysis of the control and rotenone-treated *Drosophila* and validation of the models. (**A**) Experimental design of this study to show different groups of models, including control *Drosophila* (CTRL), Parkinson’s *Drosophila* (PD), control *Drosophila* with FMT from Parkinson’s *Drosophila* (CWFP), control *Drosophila* without FMT from Parkinson’s *Drosophila* (CWOFP), Parkinson’s *Drosophila* with FMT from control *Drosophila* (PWFC), and Parkinson’s *Drosophila* without FMT from control *Drosophila* (PWOFC). (**B**) Survival curves of CTRL and PD flies within 7 days (*n* = 80), significant differences were determined by log-rank test (*P* < 0.0001). (**C**) Negative geotaxis assays to show locomotor deficits of flies exposed to rotenone. Three replicates (20 flies in each replicate) were included in each timepoint and average numbers of flies were used for statistical analysis. (**D**) Relative expression of PD-related marker genes in CTRL and PD flies. Flies (male to female as 1:1) were used in the survival and locomotion assay. Significant differences are determined by the two-way ANOVA followed by Sidak’s post-hoc test. *P < 0.05, ***P < 0.001.

To dissect the relationship between the gut microbiota and PD, we first constructed the PD models by administering rotenone to *Drosophila melanogaster* for 7 days to obtain two groups of fly samples including Parkinson’s *Drosophila* (PD) and control *Drosophila* (CTRL). PD models were validated through phenotypic observation and molecular testing, then we performed cross-colonization experiments (Fig. 1A). The PD flies and control flies were subjected with or without FMT for another 7 days. After cross-colonization experiments (day 14), we harvested flies from each treatment group, including control *Drosophila* with FMT from Parkinson’s *Drosophila* (CWFP), control *Drosophila* without FMT from Parkinson’s *Drosophila* (CWOFP), Parkinson’s *Drosophila* with FMT from control *Drosophila* (PWFC), and Parkinson’s *Drosophila* without FMT from control *Drosophila* (PWOFC).

The survival curves were significantly different between the PD and CTRL groups (Log-rank test, *P* < 0.0001). CTRL *Drosophila* had a longer lifespan than PD *Drosophila* (Fig. 1B). Moreover, PD flies exhibited significant motor deficits. In the negative geotaxis assays, PD flies seemed not to be able to coordinate the legs and tended to stay at the bottom of the column. Statistical analysis showed that the locomotor activity of PD flies was significantly affected (Fig. 1C). Rotenone is a neurotoxin that damages mitochondria, Mul1 and Park are two major E3 ubiquitin ligases controlling the stability of mitochondria (16). Also, PD is a neuro-degenerative disease caused by the severe deficiency of Dopamine (DA) in the substantia nigra of striatum. Tyrosine hydroxylase (TH) is a key enzyme in the DA biosynthesis pathway (17). Quantitative RT-qPCR results showed that the expression levels of these marker genes in PD *Drosophila* were significantly lower than those in CTRL *Drosophila* (Fig. 1D). Thus, our results demonstrated that rotenone-induced PD flies could develop typical symptoms with marker genes changed, suggesting that our PD models were successfully constructed.

### The effect of PD-associated gut microbiota on microbial composition of *Drosophila*

**Fig 2:**
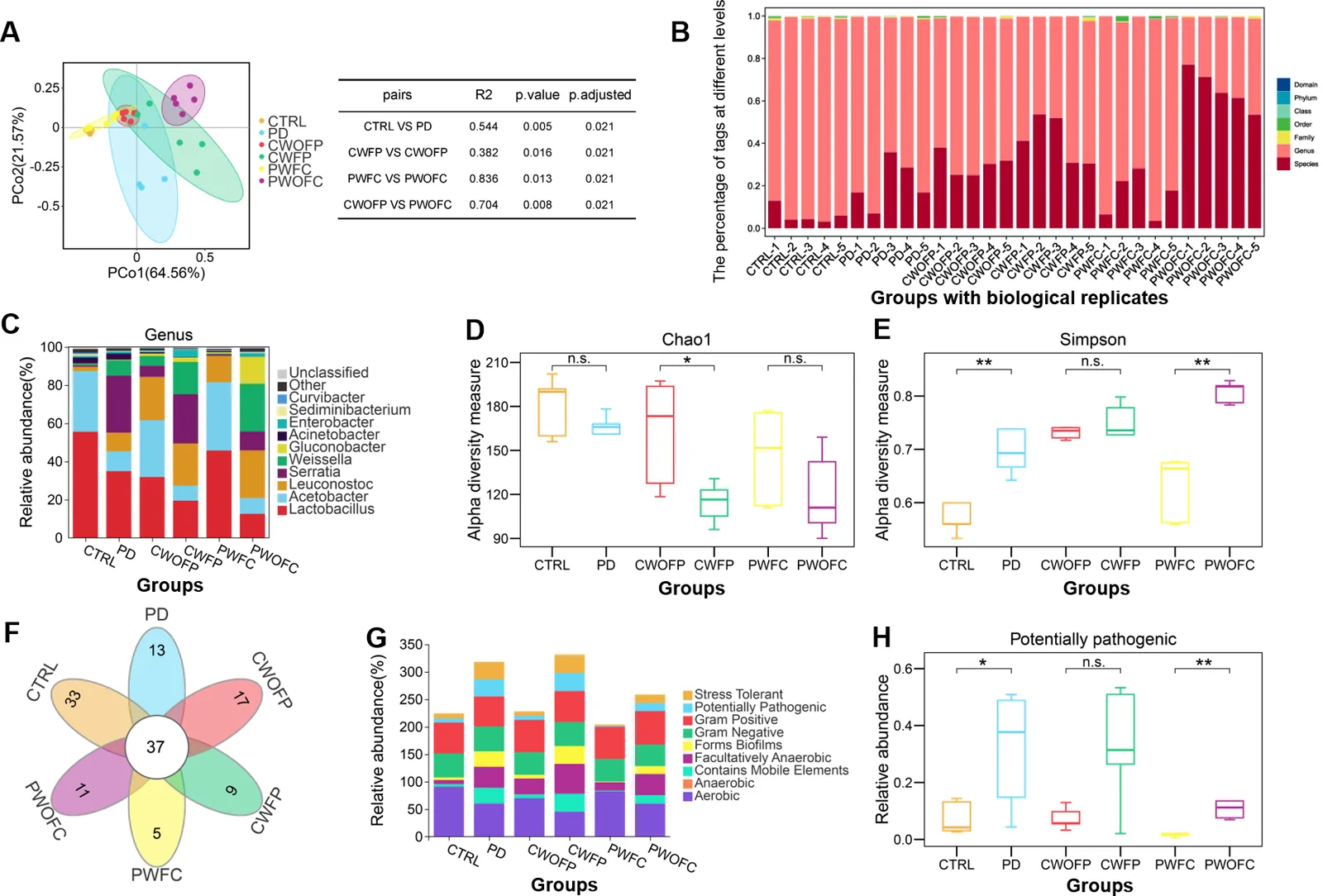
FMT significantly changed the composition of gut microbiota in each group *Drosophila*. (**A**) Analysis of β-diversity of gut microbiota in *Drosophila*. PCoA was used to calculate the distance between samples, and the clusters between groups were tested with PERMANOVA. Each point represented a sample, *n* = 5. (**B**) According to the species annotation information of OTUs/ASVs, the number of tags sequences of each sample at each classification level was counted. The vertical axis represented the percentage of each sample in the sequence at each classification level. (**C**) Gut microbiota analysis of bacterial structure in six groups at the genus level. (**D**) Chao1 α-diversity index of grouped data (*n* = 5). (**E**) Simpson α-diversity index of grouped data (*n* = 5). (**F**) Species Venn diagrams of six groups at the genus level. (**G**) The phenotypic distribution of each group analyzed by BugBase. (**H**) Relative abundance of pathogenic microbiota (*n* = 5). Control *Drosophila* (CTRL) and Parkinson’s *Drosophila* (PD) are flies before cross-colonization experiments (day 7). Control *Drosophila* with FMT from Parkinson’s *Drosophila* (CWFP), control *Drosophila* without FMT from Parkinson’s *Drosophila* (CWOFP), Parkinson’s *Drosophila* with FMT from control *Drosophila* (PWFC), and Parkinson’s *Drosophila* without FMT from control *Drosophila* (PWOFC) are flies after cross-colonization experiments (day 14). Significant differences are determined by the Wilcoxon test. *P < 0.05, **P < 0.01.

After the phenotypic confirmation of PD model and cross-colonization experiments, we performed microbial composition analysis using 16S rDNA sequencing of samples from six groups, and compared the changes of the microbiome composition between each group. Principal coordinate analysis (PCoA) of species abundance based on operational taxonomic unit (OTU) showed significant separation of samples from six groups of *Drosophila*, including the comparisons of CTRL vs PD, CWFP vs CWOFP, PWFC vs PWOFC, and CWOFP vs PWOFC (Fig. 2A). In order to better illustrate the effect of FMT on the gut microbiota of CTRL and PD groups, the microbiome profile of each sample was represented by a tags sequence. The global percent of tags were different at the genus and species levels for comparisons of CTRL vs PD or CWFP vs CWOFP or PWFC vs PWOFC (Fig. 2B). We counted the top 10 abundant microbial compositions in each group using stacked bar charts (Fig. 2C; Table S3). Compared to the CTRL group, PD groups had reduced *Lactobacillus* and *Acetobacter*. In addition, we found that *Serratia* and *Weissella* were mainly distributed in PD-associated groups, including PD group, CWFP group, and PWOFC group (Fig. 2C). We also presented the proportion of species in each group at the level of phylum, class, order, family, and species, respectively (Fig. S1). Together, the comparisons of CWFP vs CWOFP and PWFC vs PWOFC indicated that FMT significantly affected the global microbial composition in CTRL and PD *Drosophila*.

To confirm the effect of FMT on microbial diversity in CTRL and PD *Drosophila*, we analyzed the α-diversity of gut microbiota of six groups in-depth. Dilution curves of α-diversity indexes indicated that our microbial sequencing data were reliable (Fig. S2). Further analysis on α-diversity indexes and rank abundance curves showed that species diversity was different in the comparisons of CWFP vs CWOFP or PWFC vs PWOFC (Fig. 2D and E; Fig. S3). Venn diagram showed that there were 37 shared species in the six groups at the genus level (Fig. 2F), and the CTRL group had the highest number of unique species (Fig. 2F; Fig. S4A). Importantly, FMT with gut microbiota from PD group increased the abundance of *Serratia* and *Weissella* in CWFP group, and FMT with gut microbiota from CTRL group increased the abundance of *Acetobacter* and *Lactobacillus* in PWFC group (Fig. 2C; Fig. S4B). The taxa that most likely explain the differences between groups were defined by LEfSe, which showed that *Lactobacillaceae*, *Acetobacteraceae,* and *Alphaproteobacteria* were significantly enriched in CTRL group, while *Serratia*, *Enterobacteriaceae,* and *Weissella* exhibited relatively higher abundance in the PD group (Fig. S4C). BugBase analysis of phenotype abundance showed that PD group has significantly higher abundance of pathogenic microbiota than CTRL group (Fig. 2G and H). Phenotypic abundance in CWFP group was similar to PD group, while PWFC group has significantly lower abundance of pathogenic microbiota than PWOFC group, suggesting that FMT experiments were successful and had obviously influenced the phenotype abundance in CTRL and PD *Drosophila*.

### The impact of PD-associated microbiota on *Drosophila* transcriptome

**Fig 3:**
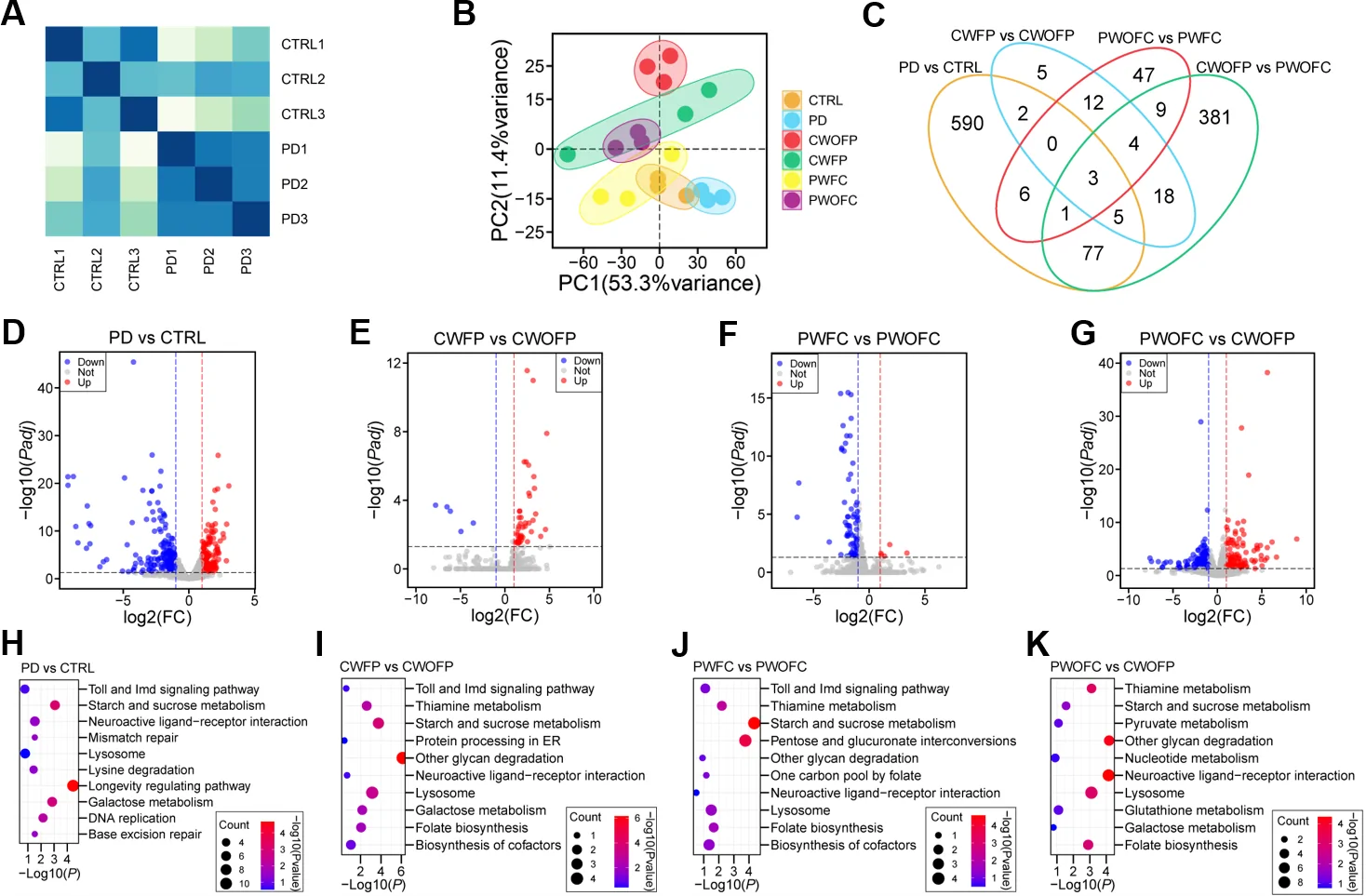
RNA sequencing analysis of *Drosophila* samples before and after FMT experiments. (**A**) Pearson correlation heatmap of RNA-seq data from CTRL and PD groups. (**B**) PCA of RNA-seq data showed the separation of six groups, *n* = 3. (**C**) Venn diagram showed the numbers of DETs between four pairs of comparisons. (**D**) Volcano plot showed DETs between CTRL group and PD group. (**E**) Volcano plot showed DETs between CWFP group and CWOFP group. (**F**) Volcano plot showed DETs between PWFC group and PWOFC group. (**G**) Volcano plot showed DETs between PWOFC group and CWOFP group. (**H**) Enrichment analysis of KEGG pathway between CTRL group and PD group. (**I**) Enrichment analysis of KEGG pathway between CWFP group and CWOFP group. (**J**) Enrichment analysis of KEGG pathway between PWFC group and PWOFC group. (**K**) Enrichment analysis of KEGG pathway between PWOFC group and CWOFP group. The x-axis represented enrichment factors and the y-axis represented different pathways of biological processes.

After determining the effects of FMT with PD-associated microbiota on different models, we further performed RNA sequencing using mRNAs from six groups of flies. We first drew the heatmap according to Pearson correlation coefficient of gene expression, and the results showed that the expression levels of transcripts were significantly different in the CTRL group compared with the PD group (Fig. 3A). Principal component analyses (PCAs) of transcriptome data of six groups were performed, and the results suggested that there were separation between the groups (Fig. 3B). We analyzed the differentially expressed transcripts (DETs) of six groups, and found 684 DETs between CTRL group and PD group, 49 DETs between CWFP group and CWOFP group, 82 DETs between PWFC group and PWOFC group, and 498 DETs between PWOFC group and CWOFP group (Fig. 3C; Table S2). Among these DETs, we further analyzed the upregulated and downregulated transcripts between different groups (Fig. 3D through G).

KEGG analysis was performed to identify signaling pathways enriched for DETs with or without FMT experiments, and the results showed that various signaling pathways were enriched for different groups (Fig. 3H through K). Importantly, neuroactive ligand-receptor interaction, lysosome, and diverse metabolic pathways were enriched for four groups of comparisons (PD vs CTRL, CWFP vs CWOFP, PWFC vs PWOFC, and PWOFC vs CWOFP). Since all compared *Drosophila* were grown at the same development stage and treated with or without FMT, the observed differences in enriched pathways were proposed to derive from PD-associated microbiota and these common pathways may be associated with PD pathogenesis regulated by gut microbiota.

### Correlations analysis of functional pathways influenced by PD-associated gut microbiota

**Fig 4:**
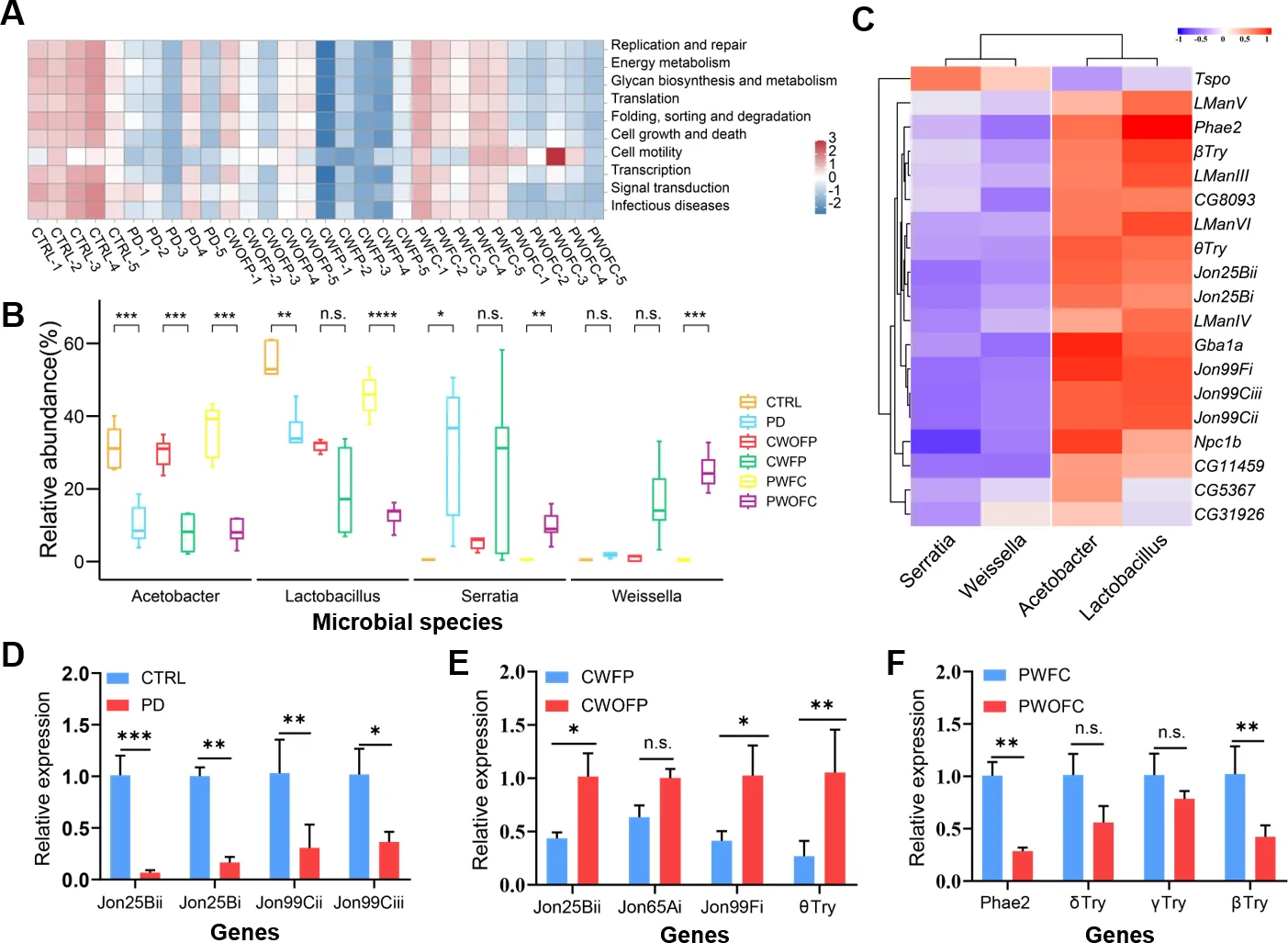
Correlations analysis of functional pathways influenced by PD-associated gut microbiota and RT-qPCR validation of genes. (**A**) Functional prediction heatmap with PICRUSt2. Vertical axis indicated functional classification, horizontal axis indicated samples, and the color indicated the richness of the function. (**B**) Indicator species analysis at genus level of six groups. (**C**) Correlation analysis of the four gut microbes with DETs, which are representative genes from pathways of “neuroactive ligand-receptor interaction” and “lysosome” in Fig. 3. (**D**) Relative expression of transcripts encoding neuroactive ligand receptor interactions in CTRL group and PD group. (**E**) Relative expression of transcripts encoding neuroactive ligand-receptor interaction in CWFP group and CWOFP group. (**F**) Relative expression of transcripts encoding neuroactive ligand-receptor interactions in PWFC group and PWOFC group. Significant differences were determined by the unpaired Student’s *t*-test. *P < 0.05, **P < 0.01, ***P < 0.001.

We predicted functional pathways across microbial samples using PICRUSt2, and found that various pathways were decreased in PD group than CTRL group, such as replication and repair, energy metabolism, cell growth and death, glycan biosynthesis and metabolism, etc. After FMT, the abundance of these pathways was lower in CWFP group than CWOFP group. Similarly, the abundance of these pathways was lower in PWOFC than PWFC group (Fig. 4A). Statistical analysis was performed on species at genus level among the six groups, and we found that the abundance of *Lactobacillus*, *Acetobacter*, *Serratia,* and *Weissella* was significantly different in most comparisons, including PD vs CTRL, CWFP vs CWOFP, and PWFC vs PWOFC (Fig. 4B). For example, the abundance of *Acetobacter* was lower in PD group than CTRL group. After cross-colonization FMT experiments (without rotenone in the medium), the abundance of *Acetobacter* in CWFP group became lower than CWOFP group, and the abundance of *Acetobacter* in PWFC group became higher than PWOFC group.

We performed correlations analysis between the four gut microbes and DETs in six groups, and the results showed that most of the DETs were positively correlated with *Acetobacter* and *Lactobacillus*, and negatively correlated with *Serratia* and *Weissella* (Fig. 4C). Moreover, we used Cytoscape software to analyze the correlation of DETs from neuroactive ligand-receptor interaction and lysosome pathways with the four gut microbes (Fig. S5), and the results showed that the DETs were correlated with the four gut microbes (*Acetobacter, Lactobacillus*, *Serratia,* and *Weissella*).

To verify our RNA-seq data, we performed RT-qPCR experiments and examined some DETs in neuroactive ligand-receptor interaction pathway, which are the most significantly enriched pathway between the PD and the CTRL group. The results showed that the expression levels of *Jon25Bi*, *Jon25Bii*, *Jon99Cii,* and *Jon99Ciii* were significantly downregulated by rotenone exposure (Fig. 4D). FMT significantly reduced the expression of *Jon25Bii*, *Jon99Fi,* and *θTry* in the CWFP group compared to the CWOFP group (Fig. 4E). The genes of *βTry* and *Phae2* were significantly upregulated in PWFC group compared to the PWOFC group (Fig. 4F). Overall, RT-qPCR results of these DETs were generally similar to their expression patterns in RNA-seq data, indicating the validity and reliability of our RNA-seq data sets. Together, the results suggested that FMT with PD-associated gut microbiota could affect gene expression in *Drosophila*.

### PD-associated gut microbiota from clinical patients influences microbial structure and diversity in *Drosophila*

**Fig 5:**
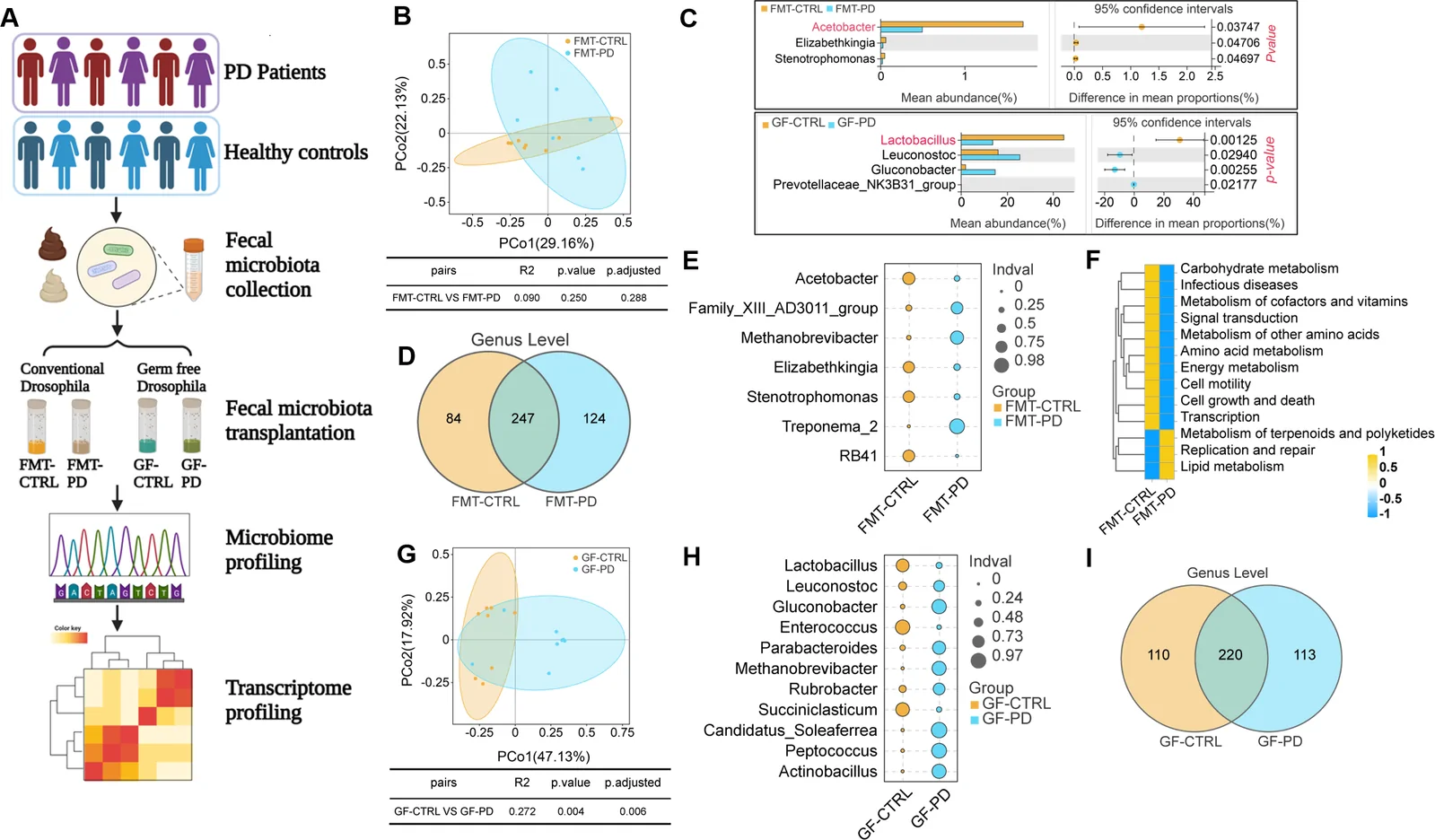
Microbial structure and diversity in *Drosophila* colonized with fecal samples from clinical patients. (**A**) Schematics diagram showing experimental design of this study. (**B**) Analysis of β-diversity of gut microbiota in FMT-CTRL and FMT-PD groups from conventional *Drosophila*. PCoA was used to calculate the distance between samples, and the clusters between groups were tested with PERMANOVA. Each point represents a sample, *n* = 7 or 8. (**C**) Welch’s *t*-test showing differences in species abundance for conventional *Drosophila* and germ-free *Drosophila*, *P < 0.05. (**D**) Venn diagram indicating the numbers of species in two groups. (**E**) Indicator value (Indval) of species in each group at the genus level. (**F**) Functional prediction heatmap with PICRUSt2. (**G**) Analysis of β-diversity of gut microbiota in GF-CTRL and GF-PD groups from germ-free *Drosophila*. PCoA was used to calculate the distance between each sample. Each point represents a sample, *n* = 8. (**H**) Indicator value (Indval) of species in each group at the genus level. (**I**) Venn diagram indicating the numbers of species in two groups.

To further confirm whether gut microbiota-mediated gene expression changes derived from rotenone-induced PD model can be resembled by clinical PD-associated gut microbiota, we collected fecal samples from clinical PD patients for FMT experiments. The fecal samples were collected from six PD patients and six healthy controls with male to female ratio as 1:1. The fecal samples with gut microbiota were mixed as fecal suspensions and firstly colonized into conventional *Drosophila*. The effects of gut microbiota from PD patients on *Drosophila* were evaluated by 16S rDNA sequencing and transcriptome sequencing (Fig. 5A).

The clusters of FMT-CTRL *Drosophila* and FMT-PD *Drosophila* appeared to be sparse by PCoA (Fig. 5B). However, the most significantly different genus was *Acetobacter* (Fig. 5C), which was also the different genus in a rotenone-induced PD model. Venn diagram was made to define the core microbiome at the genus level, and we identified 124 species specifically in FMT-PD *Drosophila* (Fig. 5D). We calculated the indicator value of each species in each group at the genus level and confirmed that *Acetobacter* was one of the indicator species between FMT-CTRL *Drosophila* and FMT-PD *Drosophila* (Fig. 5E). We analyzed functional pathways with PICRUSt2, and found that various metabolism pathways were different between FMT-CTRL *Drosophila* and FMT-PD *Drosophil*a (Fig. 5F). The microbial composition profiles for PD patients and healthy controls before FMT were also analyzed (Fig. S6), and the indicator values showed that *Acetobacter* was one of the indicator species. The alpha diversity of microbial composition for patient samples was insignificant, which may be due to the limited number of clinical samples.

The fecal samples from PD patients and healthy controls were also colonized into germ-free *Drosophila* followed by 16S rDNA sequencing (Fig. 5G). The indicator values of each species in each group at the genus level showed that *Lactobacillus* was one of the indicator species between GF-CTRL *Drosophila* and GF-PD *Drosophila* (Fig. 5H). In germ-free *Drosophila*, we identified 113 species specifically in GF-PD *Drosophila* (Fig. 5I). Together, our FMT experiments with PD patients in conventional *Drosophila* and germ-free *Drosophila* support our conclusion that *Acetobacter* and *Lactobacillus* are negatively associated with PD.

### PD-associated gut microbiota from clinical patients influences *Drosophila* metabolic signaling pathways

**Fig 6:**
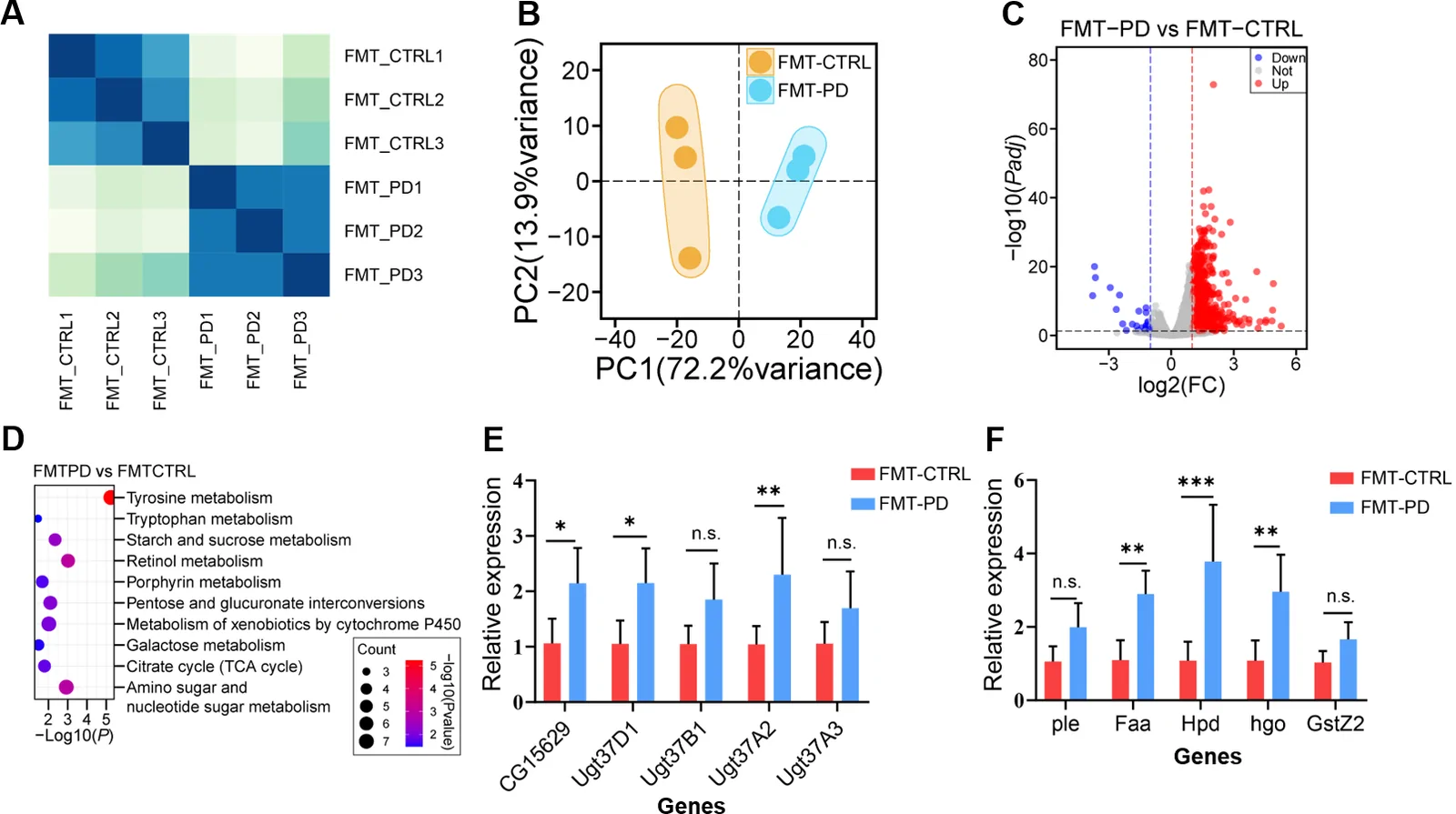
Analysis of *Drosophila* transcriptome transplanted with PD-associated gut microbiota from clinical patients. (**A**) Pearson correlation heatmap of RNA-seq data from FMT-PD group and FMT-CTRL group, *n* = 3. (**B**) PCA of RNA-seq data from FMT-PD group and FMT-CTRL group, *n* = 3. (**C**) Volcano plot showing differentially expressed genes of *Drosophila* transplanted with PD-associated gut microbiota. (**D**) Enrichment analysis of the KEGG pathway of differentially expressed genes. (**E**) Relative expression level of genes associated with tyrosine metabolism in RT-qPCR. (**F**) Relative expression level of genes associated with retinol metabolism in RT-qPCR. Significant differences are determined by the unpaired Student’s *t*-test. *P < 0.05, **P < 0.01, ***P < 0.001.

Next, we investigated the effects of PD-associated gut microbiota on host gene expression. We first analyzed the expression patterns between FMT-CTRL *Drosophila* and FMT-PD *Drosophil*a groups by drawing the heatmap according to gene expression profiles. The results showed that the expression patterns were significantly different between FMT-CTRL *Drosophila* and FMT-PD *Drosophil*a (Fig. 6A). PCA of RNA-seq data showed that three biological replicates from two groups were well clustered, while samples from FMT-CTRL *Drosophila* and FMT-PD *Drosophila* were clearly separated (Fig. 6B). Among these DETs, 858 transcripts were upregulated and 27 transcripts were downregulated when comparing FMT-CTRL *Drosophila* to FMT-PD *Drosophila* (Fig. 6C).

KEGG analysis was performed and the results revealed that differentially expressed transcripts were enriched in metabolism pathways, including tyrosine metabolism, retinol metabolism, starch and sucrose metabolism, galactose metabolism, and porphyrin metabolism (Fig. 6D). To verify the results of RNA-seq data, we selected differentially expressed genes enriched for tyrosine metabolism (Fig. 6E) and retinol metabolism (Fig. 6F) for RT-qPCR validations. The results showed that 6 out of the 10 upregulated transcripts in RNA-seq data were also upregulated in RT-qPCR experiments, indicating the validity and reliability of our RNA-seq data sets. Collectively, our *Drosophila* FMT experiments with fecal samples from clinical PD patients and rotenone-induced PD models revealed that PD-associated gut microbiota can influence the expression of genes enriched in metabolic signaling pathways.

## DISCUSSION

Multiple studies have also proven that the pathogenesis of the chronic PD is closely related to gut microbiota dysbiosis (18 – 20). Better understanding of the interaction between gut microbiota and PD may provide novel insights into PD pathogenesis and help develop new therapeutic strategies. *Drosophila melanogaster*, one of many species of fruit fly, has been used as an optimal research model in the field of genetic engineering. In recent years, *Drosophila* has gained attention as an experimental animal for gut microbiota research in neurodegenerative diseases (21). In this study, we established PD *Drosophila* models by rotenone and investigated the role of gut microbiota in the pathogenesis of PD. The result showed that PD flies exhibited reduced lifespan and climbing and flight defects, meanwhile the expression levels of marker genes in PD *Drosophila* were significantly lower than those in CTRL *Drosophila*, which was consistent with the previous report. Based on 16S rDNA sequencing, we found that the species diversity in the PD group was much lower than that of the CTRL group. Our results demonstrated that the *Drosophila* PD model was successfully constructed in our study.

FMT with gut microbiota has been emerging as a promising therapy in various diseases (22, 23). In addition, one case report suggested that FMT can protect the gastrointestinal dysfunctions and motor disorders in a PD patient (24). To further assess the effects of FMT treatment on PD, we treated *Drosophila* with FMT and found that the gut microbiota of flies could be clearly separated after FMT. We counted the top 10 abundant bacteria at the genus level, and we found that *Lactobacillus* is the most widely distributed gut microbiota followed by *Acetobacter*. It has been demonstrated that the gut microbiota can regulate many biological processes by influencing gene expression in insects (25, 26). Therefore, we performed transcriptomic analysis by RNA-seq to further identify the genes involved in biological processes influenced by gut microbiota. We found that the expression levels of transcripts were significantly different in the CTRL group compared with the PD group. It is noteworthy that DETs were enriched in neuroactive ligand-receptor interaction, lysosome, and diverse metabolic pathways in all pairs of comparisons (PD vs CTRL, CWFP vs CWOFP, and PWFC vs PWOFC). Notably, all comparisons generally showed a consensus of difference in microbes and gene expression. Our results from rotenone-induced PD models were supported by FMT experiments with fecal samples from clinical PD patients. In *Drosophila* transplanted with fecal samples from PD patients, *Acetobacter* and *Lactobacillus* were associated with differentially expressed genes enriched in diverse metabolic pathways. Importantly, we found that starch/sucrose/galactose metabolism pathways were enriched in both rotenone-induced PD models and FMT with clinical samples. Since rotenone was not included in the fly medium when we performed cross-colonization FMT, so presumably the observed differences in microbes and gene expression from comparisons of CWFP vs CWOFP or PWFC vs PWOFC were mainly from the effect of FMT experiments. However, the side effect from rotenone in our study could not be completely excluded and should be considered in future work, since rotenone-induced PD models and FMT with clinical samples still had many different functional pathways. Taken together, our FMT experiments with gut microbiota from both rotenone-induced PD samples or clinical patients samples suggested that PD-associated gut microbiota can impact host gene expression in *Drosophila*.

As previously indicated, the simplicity of fruit flies provides a prominent advantage for FMT (27, 28), and the simple gut microbiota of *Drosophila* can help us understand the impact of gut microbiota on host physiology. Although *Drosophila* has orthologs for about 75% of human disease-related genes and the fly’s nervous system shares basic structure and functional features with humans (29), there are still differences in gut microbiota compositions and gene expression patterns between *Drosophila* and humans. In our study, we found that many gut microbiota species identified from human fecal samples were hard to be colonized in *Drosophila*. In both rotenone-induced PD *Drosophila* and *Drosophila* colonized with PD patient samples, the abundance of the two gut bacteria *Lactobacillus* and *Acetobacter* were obviously changed. In addition, we found that conventional *Drosophila* and germ-free *Drosophila* have different altered gut microbiota after FMT experiments. While the results from different contexts suggested that both *Acetobacter* and *Lactobacillus* were associated with PD pathogenesis, the genera *Serratia* and *Weissella* could also be related to PD pathogenesis. For instance, the genus *Serratia* belongs to the family *Enterobacteriaceae* and is now recognized as an important pathogen capable of causing many infections (30). Whether the genera *Serratia* and *Weissella* are involved into the pathogenesis of PD requires further investigations. In addition, our experiments included the same numbers of male and female *Drosophila*. Since male and female flies have different mechanisms for physiological processes (31, 32) and female flies are larger than male flies, the results of microbiome and transcriptome may have gender bias. RNA transcript abundance results could be biased toward female flies, and qPCR/RNA-seq experiments using single gender of flies are warranted to separate the effects of male and female flies. In the future, individual strain colonization should be carried out in a specific gender of *Drosophila* to explore the mechanism of gut microbiota on PD. Future work should also include more controls to rule out the side effects from other factors, such as non-FMT *Drosophila* controls when using clinical samples for colonization.

In summary, our results revealed the effect of gut microbiota on PD *Drosophila* by FMT and suggested that reduced *Acetobacter* and *Lactobacillus* were associated with PD pathogenesis. Transcriptome data analysis demonstrated that PD-associated gut microbiota could affect *Drosophila* gene expression. Taken together, our findings provide fundamental information for the impact of gut microbiota on transcriptomes in PD revealed by FMT. We anticipate this study can facilitate the understanding of the mechanism underlying PD treatment with FMT in clinical practice.

## MATERIALS AND METHODS

### Construction of rotenone-induced PD model in Drosophila

*Drosophila* melanogaster w^1118^ was reared at 25℃ under 12-hour light/12-hour dark cycles on yeast-glucose medium (1 L water, 100 g yeast, 100 g glucose, 1.2% agar, and 0.1% potassium sorbate) (33, 34). To construct PD model, we first dissolved rotenone (C_23_H_22_O_6_, #83–79-4, Dingguo Biotechnology Co., Ltd.) in DMSO as a stock solution (25 mM). Adult flies (day 7 post-eclosion) were cultured in a yeast-glucose-based medium supplemented with 70 µL of rotenone stock solution or DMSO for 7 days according to previous literature (16). For the control, *Drosophila* was cultured in a yeast-glucose-based medium containing only DMSO (#60313ES60, Yeasen). Each bottle contains the same number of male and female flies. Rotenone-containing food was freshly prepared, and the food should be changed every 2 days to ensure the efficacy.

### Survival and locomotion assays

For the phenotypic experiments, adult flies were transferred to yeast-glucose medium with either DMSO (CTRL flies) or 250 µM rotenone dissolved in DMSO (PD flies) for 7 days. In order to validate the PD *Drosophila* model, 80 flies subdivided into 4 replicates (20 flies in each replicate) and were used for PD and CTRL, respectively. For the evaluation of the drug feeding effect, the surviving number of *Drosophila* was recorded every day. We determined locomotor ability with a negative geotaxis assay. Flies (male to female as 1:1) were anesthetized and placed in a vertical plastic column (length, 25 cm; diameter, 1.5 cm). After 30 minutes recovery from CO_2_ exposure, flies were gently tapped to the bottom of the column. Each 1 minute interval, the number of flies that climbed across a target line was recorded (35). Three replicates (20 flies in each replicate) were included in each timepoint and average numbers of flies were used for statistical analysis.

### Fecal microbiota transplantation experiments

For FMT experiments with fecal samples from rotenone-induced PD models in *Drosophila*, we first obtained rotenone-induced PD flies or CTRL flies as in the above method. After 7 days, *Drosophila* were anesthetized with CO_2_, then surface disinfected with 70% ethanol, and washed with sterile PBS (#C0221A, Beyotime) three times. PD flies or CTRL flies were ground separately in 500 µL PBS solution with steel balls to obtain gut content. Each gut content was derived from PD flies or CTRL flies with equal numbers of males and females to avoid sex bias (10 flies per replicate). For cross-colonization experiments, PD flies were then fed for another 7 days with medium containing 50 µL of gut content from CTRL flies. Similarly, CTRL flies were fed for another 7 days with medium containing 50 µL of gut content from PD flies. For other groups of *Drosophila* without FMT, the flies were continuously reared in yeast-glucose medium for another 7 days. For FMT with fecal samples from clinical PD patients, stools were taken from −80℃ and thawed in water bath at 37℃. Fecal samples from individual PD patients or normal control were then pooled and homogenized in sterile PBS with a final concentration of 200 mg feces/2 mL. Pooled samples were centrifuged at 12,000 rpm for 2 minutes, and fecal suspension were collected in sterile centrifuge tubes. FMT with patient microbiome was colonized into conventional *Drosophila* and germ-free *Drosophila*. For FMT experiments in *Drosophila*, about 200 µL of fecal suspension was added to *Drosophila* medium (without rotenone). For FMT in germ-free *Drosophila*, we first prepared germ-free *Drosophila* as previous described (36), and FMT procedures were the same as the conventional *Drosophila*. Twenty flies (male to female as 1:1) were collected on day 7 post-eclosion for 16S rDNA sequencing and RNA sequencing.

### 16S rDNA sequencing

High-throughput 16S rDNA-seq was performed in Gene Denovo Biotechnology Co. Ltd. in Guangzhou. Microbial DNA from *Drosophila* was extracted using HiPure Stool DNA Kit (#D314103, Magen, Guangzhou, China) according to manufacturer’s protocol. The 16S rDNA target region of the ribosomal RNA gene was amplified by PCR (95℃ for 5 minutes, followed by 30 cycles at 95℃ for 1 minute, 60℃ for 1 minute, and 72℃ for 1 minute and a final extension at 72℃ for 7 minutes). The primers corresponding to V3–V4 regions of 16S rRNA gene were used for PCR: 341F (CCTACGGGNGGCWGCAG) and 806R (GGACTACHVGGGTATCTAAT). PCR reactions were performed in triplicate 50 µL mixture containing 50 ng of template DNA using SMRTbell Template Library Preparation Kit (PacBio, Menlo Park, USA). Amplicons were extracted from 2% agarose gels and purified using AxyPrep DNA Gel Extraction Kit (#AP-GX-250, Axygen Biosciences) according to manufacturer’s instructions and quantified using ABI StepOnePlus Real-Time PCR System (Life Technologies). Purified amplicons were pooled in equimolar and paired-end sequenced (PE250) on an Illumina platform according to the standard protocols.

### Gut microbiome data analysis

For microbiome data analysis, the representative OTU sequences or ASV sequences were classified into organisms by a naïve Bayesian model using RDP classifier (37) (version 2.2) based on SILVA database (38) (version 132) or UNITE database (39) (version 8.0) or ITS2 database (40) (version update_2015), with the confidence threshold value of 0.8. Venn analysis was performed in R project Venn Diagram package (41) (version 1.6.16) and UpSet plot was performed in R project UpSetR package (42) (version 1.3.3) to identify unique and common species or OTUs or ASVs. Biomarker features in each group were screened by Lefse software (43) (version 1.0). OTU/ASV rarefaction curve and rank abundance curves were plotted in R project ggplot2 package (version 2.2.1). The KEGG pathway analysis of the OTUs/ASV was inferred using PICRUSt2 (44) (version 2.1.4). Microbiome composition data were analyzed using PCoA, and the clusters were tested with PERMANOVA. The software ggplot2 was used in the geom_mark_ellipse() function inside ggforce to differentiate each group of samples by drawing ellipses and colors according to the group. Based on the level of OTUs, differences in Bray-Curtis distances between groups were calculated using the R language vegan package. The pairwise Adonis test was performed based on the Bray-Curtis distance through the R language pairwiseAdonis package to determine the significance of the difference between the two groups. Chao1, Simpson (1 - ∑(Pi)^2^), and all other alpha diversity indexes were calculated in QIIME. OTU rarefaction curve and rank abundance curves were plotted in QIIME, and multivariate statistical techniques were calculated and plotted in the R project.

Microbiome phenotypes were classified using BugBase (https://github.com/knights-lab/BugBase), which was based on the Greengenes database using OUT sequences. The mapped microorganisms were divided into different categories, including Gram positive, Gram negative, biofilm forming, pathogenic, mobile element containing, aerobic, anaerobic, facultatively anaerobic, and oxidativestress tolerant. There are some overlaps among different categories, so the total proportion with all categories could be over 100%. For example, the total proportion will be 100% if only “Gram positive” and “Gram negative” categories are summarized, while Bugbase includes more than Gram positive and Gram negative categories.

For indicator species analysis, the numbers of OUT sequences were used to obtain differential OUTs in R project edgeR package, indicspecies and labdsv packages in R project were used to calculate the indicator value of each species. The indicator values were finally tested with cross-validation and presented as bubble plots.

### RNA sequencing

After total RNA was extracted, the enriched mRNAs by Oligo(dT) beads were fragmented using fragmentation buffer and reverse transcribed into cDNA with random primers using the NEBNext Ultra RNA Library Prep Kit for Illumina (#E7530, NEB) following manufacturer’s instructions. For first-strand cDNA synthesis, the fragmented and primed mRNA was reversed into cDNA using ProtoScript II Reverse Transcriptase (#M0368S, NEB) in a 20 µL reaction with the procedure of 10 minutes at 25℃ and 15 minutes at 42℃ followed by 15 minutes at 70℃. For second-strand cDNA synthesis, the Second Strand Synthesis Enzyme Mix was added to the First-Strand Synthesis reaction to react at 16℃ for 1 hour in an 80 µL reaction. Then, the cDNA fragments were purified with QiaQuick PCR Extraction Kit (#28104, Qiagen), end-repaired, poly(A) added and ligated to Illumina sequencing adapters. The ligation products were size-selected by agarose gel electrophoresis, PCR-amplified, and sequenced using Illumina HiSeq2500 by Genedenovo Biotechnology Co, Ltd (Guangzhou, China).

### Transcriptome data analysis

For data processing, quality control of sequencing data were assessed with FastQC. Adaptor sequences were trimmed by cutadapt (45) with at least 30 nt remaining length, and clean reads were mapped to the dm6 Drosophila reference genome using HISAT2 (46). FeatureCounts (47) was used to count the number of reads that mapped to a gene. Gene expression level was calculated by R package DESeq2 (48). Hierarchical clustering and principal component analysis were used to visualize the effect of different groups. The software ggplot2 was used in the geom_mark_ellipse() function inside ggforce to differentiate each group of samples by drawing ellipses and colors according to the group. Differentially expressed genes between two groups were identified by false discovery rate [adjusted *P*-value < 0.05 and log2(fold change) >1]. In order to obtain biological annotation of differentially expressed genes, gene ontology analysis was performed by clusterProfiler (49). For the correlation analysis of transcriptome and microbiome, the average value of three biological replicates corresponding to gene expression in each group was used for transcriptome data, and the average of the relative abundance of five biological replicates at the genus level in each group was used for microbiome data. Based on the above data for each group, the Spearman coefficient correlation analysis was performed using the corr.test function of the psych package in the R project.

### RNA preparation and RT-qPCR analysis

To validate the expression level of DETs from RNA-seq, RNAs were extracted from different batch of *Drosophila* adults in 1 mL Trizol with sterilized steel balls. The RNA samples were dissolved in an appropriate amount of DEPC water. NanoDrop 2000 spectrophotometer (ThermoFisher) was used to quantify the concentration and purity of total RNA at a wavelength of 260 nm. The Hifair II First Strand cDNA Synthesis Super Mix for qPCR (#11123ES60, Yeasen) was used to synthesize cDNA. Three biological replicates were used for quantitative reverse transcription PCR analysis using HieffSYBR Mixture (#11201ES08, Yeasen). The relative mRNA level of gene expression was measured with Rp49 as internal control by calculating the values of ∆Ct^Gene^/∆Ct^Rp49^ and analyzed by the 2^-∆∆Ct^ method. Primer sequences used for RT-qPCR analysis in this study are listed in Table S1.

### Statistics

Statistical analyses were performed using GraphPad Prism (version 8.0), and the log-rank test was applied in order to plot the survival curves. Student’s *t*-test was used to evaluate the difference between the climbing assay and qPCR quantitative results. *P* < 0.05 was considered as significant difference.

## Data Availability

The authors confirm that the data supporting the findings of this study are available within the article and its supplementary materials. The 16S rRNA sequencing data in this study have been deposited at NCBI Sequence Read Archive under BioProject accession number PRJNA914678. The RNA sequencing data in this study have been deposited at NCBI GEO database under accession number GSE221760.

## SUPPLEMENTAL MATERIAL

Supplemental file 1 spectrum.00176-23-s0001.docx Fig. S1 to S6.

Supplemental file 2 spectrum.00176-23-s0002.xlsx Table S1.

Supplemental file 3 spectrum.00176-23-s0003.xlsx Table S2.

Supplemental file 4 spectrum.00176-23-s0004.xlsx Table S3.

ASM does not own the copyrights to Supplemental Material that may be linked to, or accessed through, an article. The authors have granted ASM a non-exclusive, world-wide license to publish the Supplemental Material files. Please contact the corresponding author directly for reuse.
