# Supplementary material for: The impact of Parkinson’s disease-associated gut microbiota on the transcriptome in Drosophila
Source: Microbiol Spectr. 2023 Sep 27;11(5):e00176-23. doi: 10.1128/spectrum.00176-23 (PMC10581176; doi:10.1128/spectrum.00176-23)
Supplement: Supplemental file 1 — Fig. S1 to S6. [file spectrum.00176-23-s0001.docx]

**Supplementary Information**


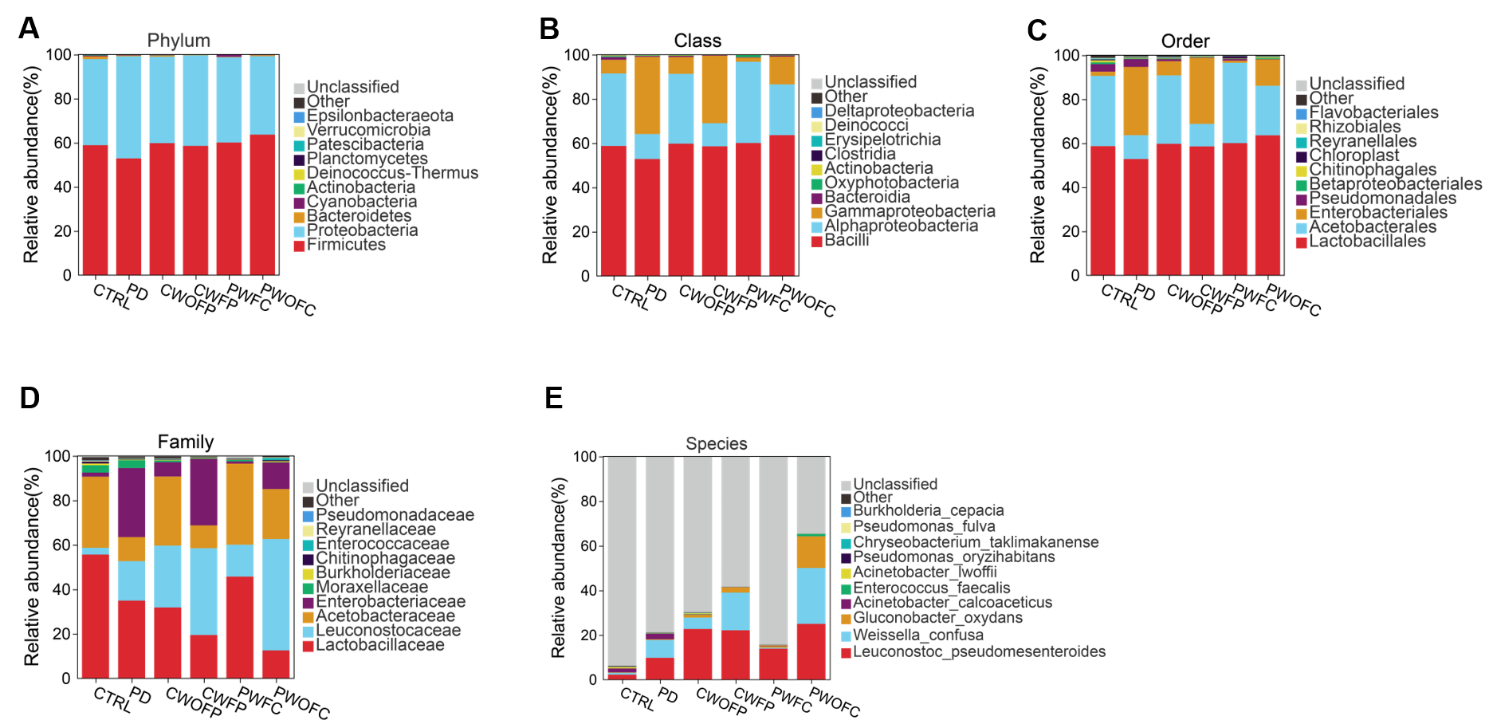


**Figure S1. Microbial analysis of samples from different groups (*n* = 5).** (A) Gut microbiota analysis of bacterial structure in six groups at phylum level. (B) Gut microbiota analysis of bacterial structure in six groups at class level. (C) Gut microbiota analysis of bacterial structure in six groups at order level. (D) Gut microbiota analysis of bacterial structure in six groups at family level. (E) Gut microbiota analysis of bacterial structure in six groups at species level. Groups include control *Drosophila* (CTRL), Parkinson's *Drosophila* (PD), control *Drosophila* without FMT from Parkinson's *Drosophila* (CWOFP), control *Drosophila* with FMT from Parkinson's *Drosophila* (CWFP), Parkinson's *Drosophila* with FMT from control *Drosophila* (PWFC) and Parkinson's *Drosophila* without FMT from control *Drosophila* (PWOFC).


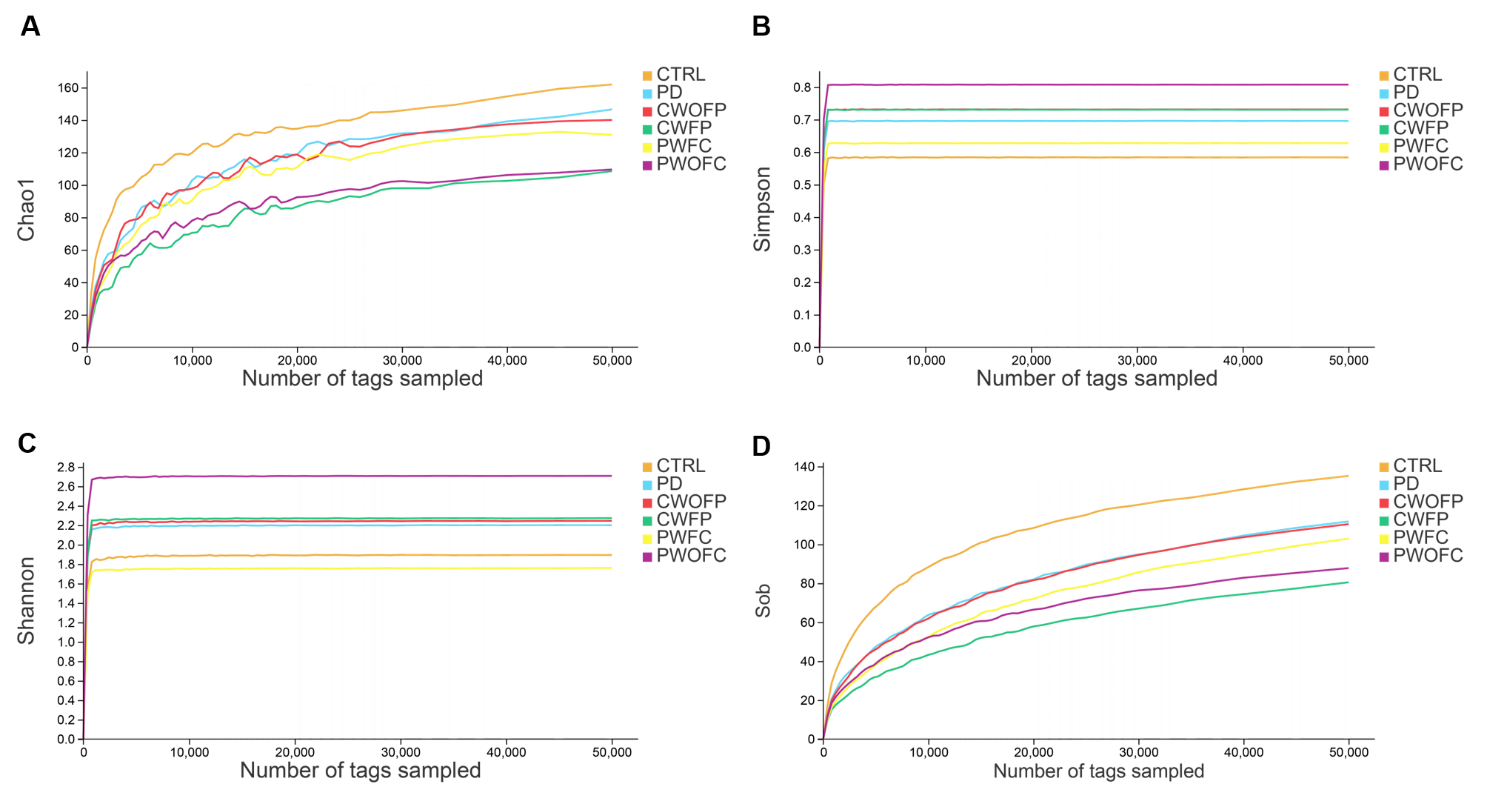


**Figure S2.** **Alpha diversity of microbial compositions before and after colonization.** (A) Dilution curves of Chao1 index in different groups. (B) Dilution curves of Simpson index in different groups. (C) Dilution curves of Shannon index in different groups. (D) Dilution curves of Sob index in different groups. The diversity of microbial composition and numbers of tags were used to plot dilution curves to represent sequencing depth of each sample. Groups include control *Drosophila* (CTRL), Parkinson's *Drosophila* (PD), control *Drosophila* without FMT from Parkinson's *Drosophila* (CWOFP), control *Drosophila* with FMT from Parkinson's *Drosophila* (CWFP), Parkinson's *Drosophila* with FMT from control *Drosophila* (PWFC) and Parkinson's *Drosophila* without FMT from control *Drosophila* (PWOFC).


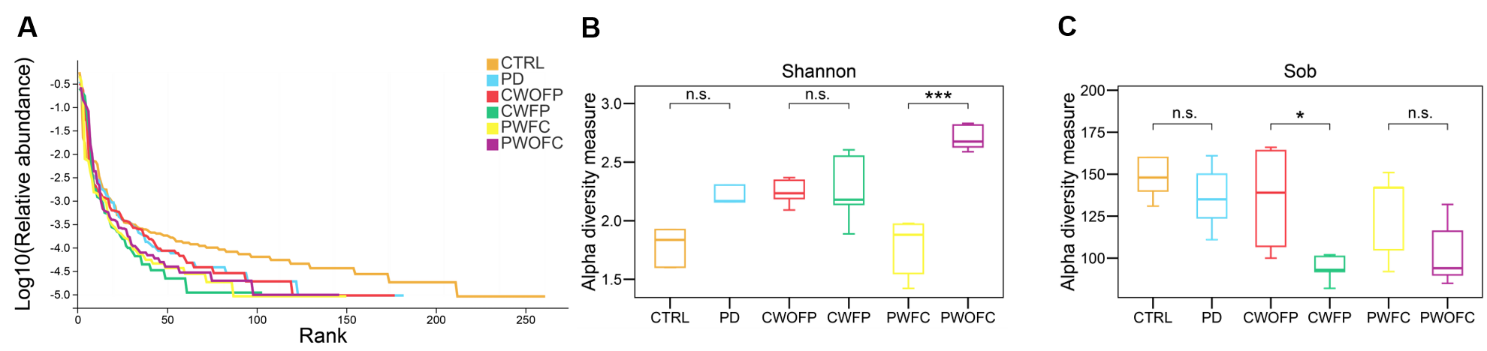


**Figure S3. Microbial compositions before and after colonization.** (A) Rank abundance curves in different groups. The x-axis represents OUT ranks, and the y-axis represents relative abundance in the form of log10. (B) Kruskal-Wallis test analysis of Simpson α-diversity index in different groups (*n* = 5). (C) Kruskal-Wallis test analysis of Sob α-diversity index in different groups (*n* = 5). Groups include control *Drosophila* (CTRL), Parkinson's *Drosophila* (PD), control *Drosophila* without FMT from Parkinson's *Drosophila* (CWOFP), control *Drosophila* with FMT from Parkinson's *Drosophila* (CWFP), Parkinson's *Drosophila* with FMT from control *Drosophila* (PWFC) and Parkinson's *Drosophila* without FMT from control *Drosophila* (PWOFC).


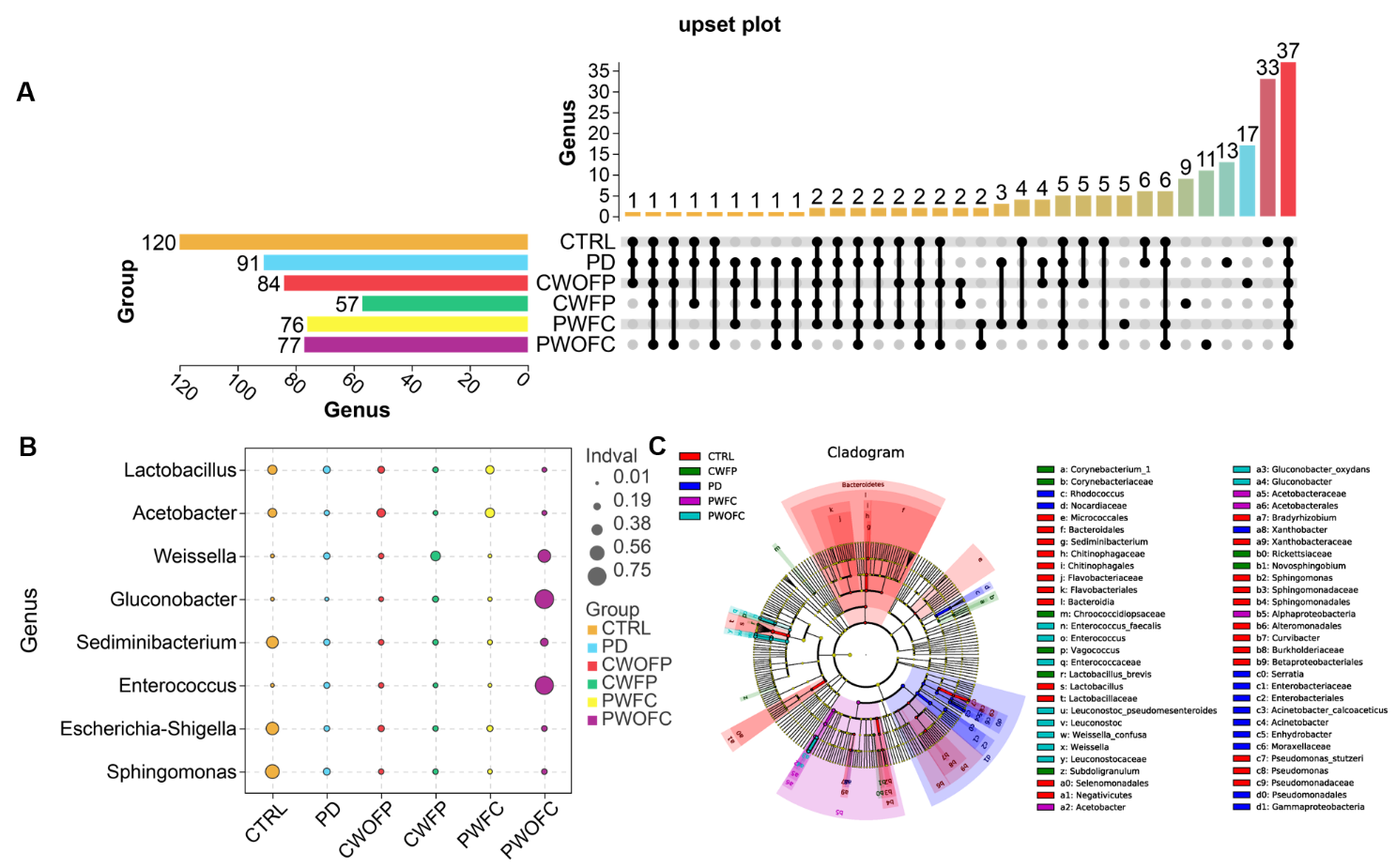


**Figure S4.** Indicator species analysis of 16S rDNA sequencing data of six groups (*n* = 5). (A) Upset graph of six groups at genus level. The horizontal columns on the left represent the numbers of species in each group. The upper right is the numbers of common/unique species represented by the corresponding dot matrix and the lower right is the dot matrix of the intersection part. The numbers in left column represent the genus identified in each group, and the numbers in upper right column represent common genus identified among all groups. In lower right, a single dot represents the unique genus and the linked dots represent genus shared among groups. (B) The abundance value in each group>0 and total proportion>0.1% of species in each group's indicator value (Indval), p-value<0.05. Indval means indicator value, the larger of the dots indicates the higher possibility of the species as the biomarker. (C) LEfSe depicting taxonomic association between microbiome communities from different groups. In the left, each circle on a different taxonomic level represents a species under that taxonomic level and the diameter of the circle is proportional to the relative abundance. In the right, the biomarkers in each group are shown, each color for the biomarkers are the same as the group.


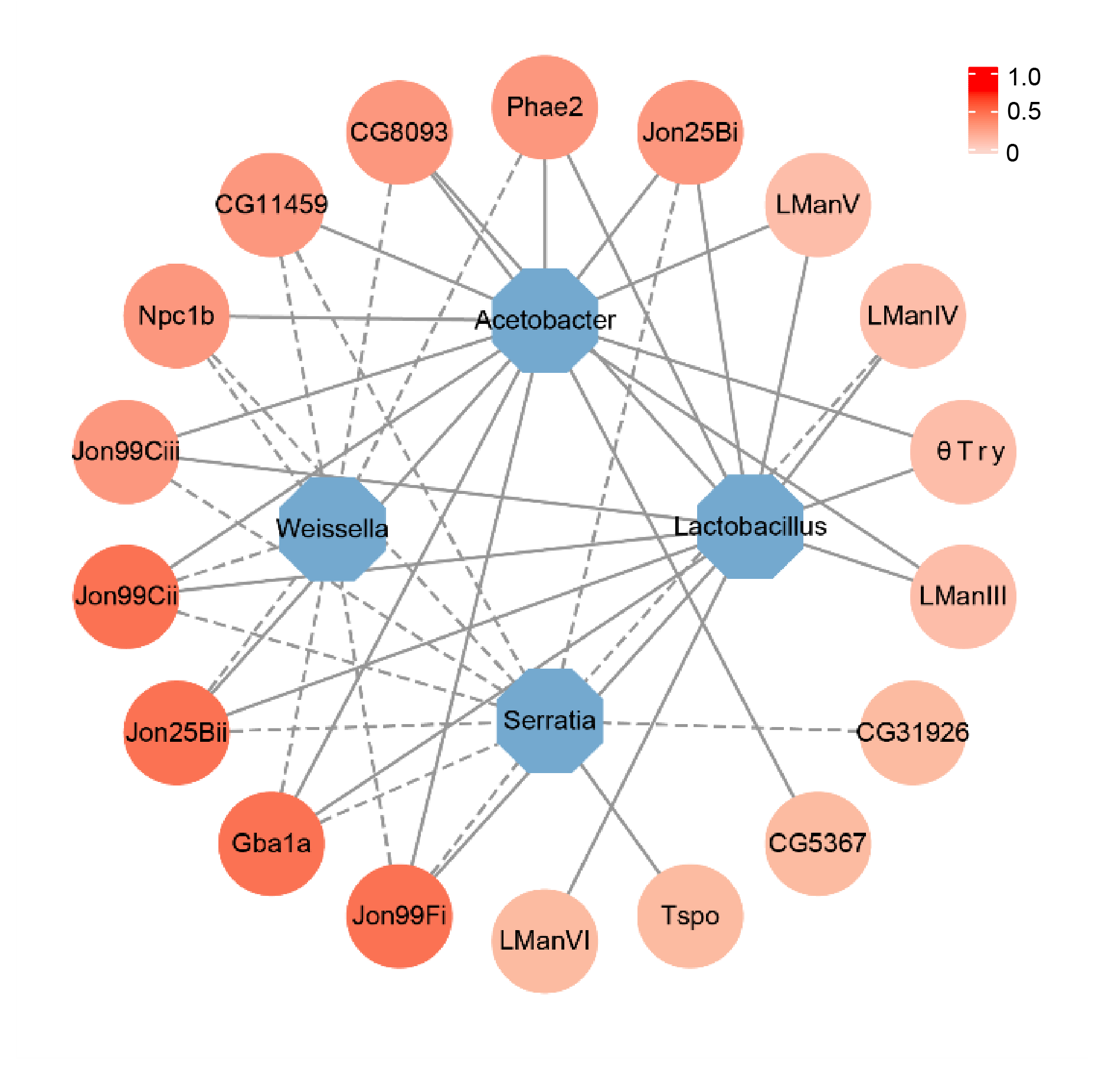


**Figure S5.** **Upregulated and downregulated transcripts were selected to analyze the correlation with the microbiome.** Networks with significant correlations were drawn using Cytoscape software. The value of correlation coefficient ranges from 0 to 1, and darker colors indicate greater correlation. The solid line and dashed line indicated positive correlation and negative correlation, respectively.


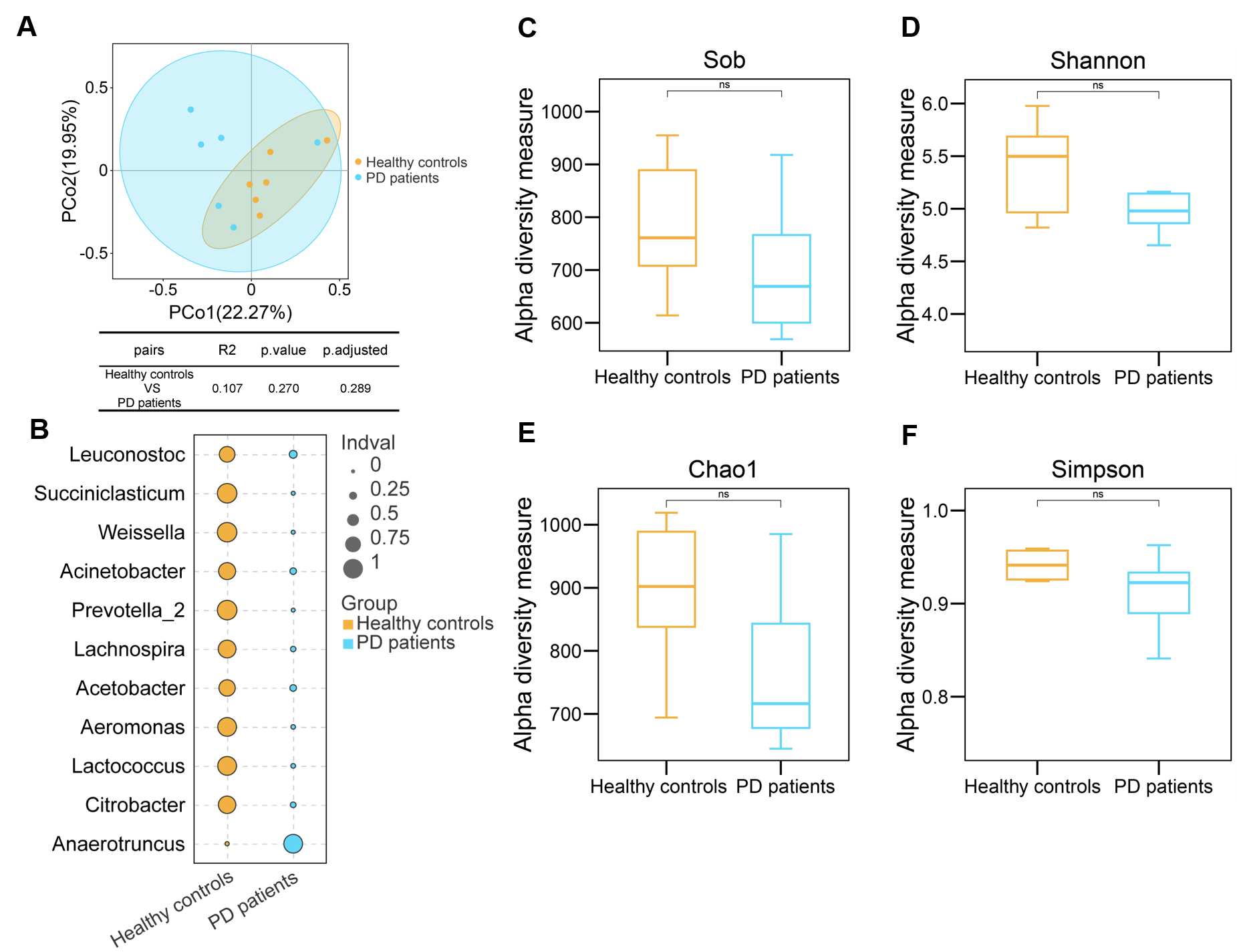


**Figure S6. Alpha and Beta diversity of human gut microbiota in each group (*n*=6).** (A) PCoA based on bray-Curtis distances at the OTU level between healthy controls and PD patients, the clusters between groups were tested with PERMANOVA. (B) Indicator value (Indval) analysis of 16S rDNA sequencing data of healthy controls and PD patients. (C) The Alpha diversity analysis of Sob index in healthy controls and PD patients. (D) The Alpha diversity analysis of Shannon index in healthy controls and PD patients. (E) The Alpha diversity analysis of Chao1 index in healthy controls and PD patients. (F) The Alpha diversity analysis of Simpson index in healthy controls and PD patients.
